# First near complete mitochondrial genome of large toothed toad, *Oreolalax major* (Anura: Megophryidae) from southwest China

**DOI:** 10.1080/23802359.2016.1144109

**Published:** 2017-01-18

**Authors:** Fuwen Liu, Yusong Liu, Yan Li, Qingyong Ni, Yongfang Yao, Huailiang Xu, Mingxian Yang, Taifu Wang, Jiacai Wang, Dingqi Rao, Mingwang Zhang

**Affiliations:** a College of Animal Science and Technology, Sichuan Agricultural University, Chengdu, Sichuan, China;; b Chengdu Leique Ecology-Environmental Protection Science and Technology Limited Company, Chengdu, Sichuan, China;; c College of Life Science, Sichuan Agricultural University, Ya’an, Sichuan, China;; d Sichuan Baodinggou Nature Reserve, Maoxian, Sichuan, China;; e Sichuan Wawushan Nature Reserve, Hongya, Sichuan, China;; f State Key Laboratory of Genetic Resources and Evolution, Kunming Institute of Zoology, Chinese Academy of Sciences, Kunming, Yunnan, China

**Keywords:** Mitochondrial genome, *Oreolalax major*, phylogenetic analysis

## Abstract

In this study, the near complete mitogenome sequence (15,469 bp) of *Oreolalax major* was determined using polymerase chain reaction (PCR). It includes 13 protein-coding genes (PCGs), 2 ribosomal RNA (rRNA) genes and 19 transfer RNA (tRNA) genes (GenBank accession number KU310894). The features of *O. major* have one more tRNA gene (*tRNA^Met^*) behind the original one before *ND2* which is similar to *Leptobrachium boringii.* Phylogenetic analyses were based on the concatenated sequences of the 13 protein-encoding genes of *O. major* and other related species.

The Large toothed toad, *Oreolalax major* (Liu & Hu 1960) belongs to family Megophryidae, genus *Oreolalax*. It is an endemic species of China, which is distributed in Hengduan Mountains of western Sichuan and southern Gansu Provinces (Fei et al. 2010; Frost 2015). But there is no mitogenome sequence of genus *Oreolalax* published until now. In this study, we sequence the mitogenome of *O. major* in order to explore the relationship among most of the genera of Megophryidae.

The *O. major* sample (Specimen voucher: 20120256) was collected from Wawushan Nature Reserve, Hongya (29°34′26.72″N, 102°56′35.73″E., elev. 1394m), Sichuan, China in June, 2015. It was fixed in 75% ethanol and deposited in the Museum of Sichuan Agricultural University. The total DNA was extracted and purified from muscle tissue using the Ezup pillar genomic DNA extraction kit (Sangon Biotech, Shanghai, China). We used twenty-three pairs of polymerase chain reaction (PCR) primers published by Zhang et al. (2013) and Kurabayashi & Sumida. (2009) to amplification and sequencing the mitogenome, internal primers were also designed when above-mentioned primers did not work. The neighbour-joining (NJ) tree based on 13 protein-coding genes (PCGs) of mitochondrial genome was reconstructed by MEGA 6.0 using Tamura–Nei model (Tamura et al. 2013).

The near complete mitochondrial genome of *O. major* is 15,469 bp in length and includes 13 protein-coding genes (PCGs), 2 ribosomal RNA (rRNA) genes and 19 transfer RNA (tRNA) genes (GenBank accession number KU310894). The features of *O. major* have one more tRNA gene (*tRNA^Met^*) behind the original one before *ND2* which is similar to *Leptobrachium boringii* (Xu et al. 2016) and most genes are encoded on H-strand except *ND6*, *tRNA^Gln^*, *tRNA^Ala^*, *tRNA^Asn^*, *tRNA^Cys^*, *tRNA^Tyr^*, *tRNA^Ser(UCN)^* and *tRNA^Glu^* genes. We regrettably did not sequence the region between 12S rRNA and *Cyt b* genes which contains 4 rRNA genes and 1 D-loop region because of difficult to amplify. There are two types of initiation codons used for 13 PCGs and most PCGs initiate from ATG, while others (*COI*, *ND5*, *ND6*) start with GTG. In addition, 5 PCGs (*ND1*, *ATP6*, *COIII*, *ND4*, *Cyt b*) terminate in incomplete stop codon T, the same number of PCGs (*ND2*, *COII*, *ATP8*, *ND3*, *ND4L*) use complete stop codon TAA, and the rest of PCGs end with AGG. The 12S rRNA and 16S rRNA are determined to be 936 bp and 1598 bp long respectively and all the tRNA genes can constitute typical cloverleaf secondary structure exclude *tRNA^Ser(AGY)^*.

The NJ analysis of the combined data set indicates that all samples of Megophryidae form a strong supported (bootstrap value = 100) monophyletic group (Figure 1) and all families (e.g. Pelodytidae/Scaphiopodidae/Pipidae) included in the analysis are monophyletic. The mitogenomic phylogenetic tree is largely consistent with the previous molecular results (Zhang et al. 2013).

**Figure 1.**
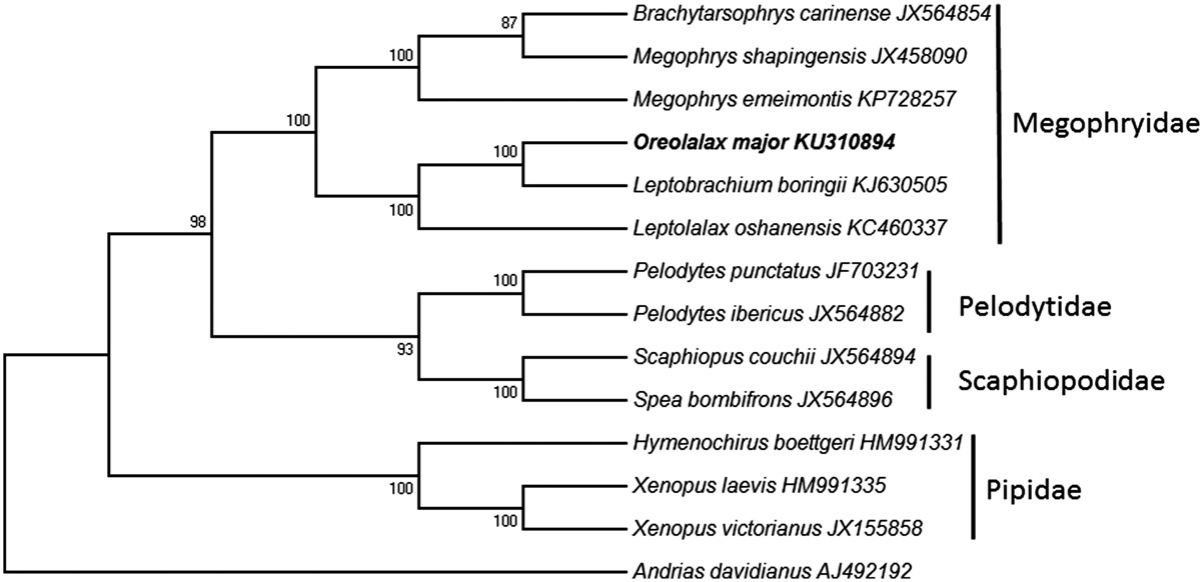
Neighbour-joining (NJ) tree was built on mitogenomic sequences of all 13 combined protein-coding genes. *Andrias davidianus* was used as outgroup. The NJ tree was reconstructed with the Tamura–Nei model, and the numbers on branches are NJ bootstrap values for 1000 replicates.
